# Interventions to improve adherence to clinical practice guidelines when treating cardiovascular disease: a systematic review

**DOI:** 10.1007/s11845-025-04057-5

**Published:** 2025-09-24

**Authors:** Sarah McErlean, Louise McCollum, Mark Ledwidge, John Broughan, Geoff McCombe, Walter Cullen, Joe Gallagher

**Affiliations:** 1 Department of General Practice, UCD School of Medicine, University College Dublin, Belfield, Dublin 4, Ireland; 2 Palms General Practice Surgery, The Avenue, Gorey, Co. Wexford Ireland; 3 School of Medicine, University of Limerick, Limerick, Ireland; 4 School of Medicine, UCD, Dublin 4, Ireland

**Keywords:** Ambulatory care, Cardiovascular disease, Guideline adherence, Implementation science, Quality improvement

## Abstract

**Background:**

Clinical practice guidelines have the potential to improve healthcare quality and safety in those with cardiovascular disease. However, guideline adherence is a challenge worldwide.

**Aims:**

We aim to determine the types and effects of interventions that target healthcare professionals to improve adherence to cardiovascular disease treatment guidelines—specifically for atrial fibrillation, ischaemic heart disease, heart failure, dyslipidaemia, and/or hypertension in ambulatory care.

**Methods:**

The following databases were searched from January 2012 to September 2023: MEDLINE, CINAHL, EMBASE,Cochrane, PsycINFO, ERIC International, Applied Social Sciences Index and Abstracts, clinicatrial.gov, and EUtrials. The search was supplemented by reviewing the reference lists of included studies, cited articles, and systematic reviews published in the field. Randomised trials, including cluster randomised trials and cross-over designs, were included. Two investigators independently extracted data and assessed study quality.

**Results:**

Forty-six studies examining the impact of healthcare-targeted interventions on improving guideline adherence were identified. Various approaches were employed, with educational interventions being the most frequently used. Given the heterogeneity in types of intervention and outcomes reported in the included trials, a narrative synthesis of the data was difficult, and meta-analysis was not planned.

**Conclusions:**

This review demonstrates that a diverse range of interventions aimed at healthcare professionals have the potential to improve adherence to CVD guidelines, but their overall effectiveness remains mixed. To narrow the gap between best practice and real-world guideline application, effective implementation strategies must become as important as guideline development itself.

**Supplementary Information:**

The online version contains supplementary material available at 10.1007/s11845-025-04057-5.

## Introduction

The prevalence of cardiovascular disease (CVD) is rising worldwide, with numbers affected nearly doubling from 271 million cases in 1990 to 523 million in 2019 [1]. As ischaemic heart disease (IHD) is the leading cause of death globally [2], focusing on the treatment and prevention of CVD is an international priority. All United Nations member states have committed to reducing premature mortality from non-communicable diseases through prevention and treatment by 2030 [3].

Guideline-adherent care has consistently been shown to improve outcomes in patients with CVD [4–7]. However, a well-established and widely discussed gap exists between evidence-based guidelines and their implementation or application [8]. Several factors contribute to this, including individual behaviour and organisational structure [8]. For guidelines to have their desired effect, they need not only rigorous development based on evidence, but also dissemination and implementation in a meaningful way [9].

Previous systematic reviews in this area published in 2014 and 2015 have shown that implementation strategies for CVD guidelines can improve guideline adherence [10, 11] and patient outcomes [10].

We aim to update the literature and evaluate what types of interventions targeting health care professionals (HCPs) are effective in improving adherence to CVD treatment guidelines for atrial fibrillation (AF), IHD, heart failure (HF), dyslipidaemia, and/or hypertension in the ambulatory care setting.

## Methods

The protocol for this systematic review was pre-registered with PROSPERO (CRD42023443990) and is reported in line with the PRISMA guidelines.

### Data sources and searches

A systematic search was conducted using terms related to ‘primary care’ or ‘ambulatory care’, ‘clinical practice guidelines’, and ‘adherence’ with the help of a medical librarian on 27th September 2023 (Appendix 1: Search Strategy).

We searched the following databases: MEDLINE, CINAHL, EMBASE,Cochrane, PsycINFO, ERIC International, Applied Social Sciences Index, and Abstracts,clinicatrial.gov and EUtrials from January 2012 to September 2023 for relevant studies that increased adherence to clinical practice guidelines for HCPs in the ambulatory care setting when treating AF, hypertension, dyslipidaemia, IHD, or HF.

In addition, we also searched reference lists of the included studies, citing articles and reference lists of published systematic reviews in the field. A bibliography of included articles was circulated to experts in the field to ensure literature saturation. Studies were limited to randomised controlled trials (RCTs).

### Inclusion criteria

-We included full-text reports, published in English since 2012.
-RCTs; including cluster randomised trials and cross-over designs were included. This restriction was to ensure that only the highest levels of evidence were included.
-Condition or domain being studied: Hypertension, dyslipidaemia, atrial fibrillation, heart failure, ischaemic heart disease.
-Participants/population: HCPs in general practice, primary care, ambulatory care, or the secondary care outpatient setting. Including primary care clinics, community health clinics, hospital outpatients, and specialist clinics.
-Intervention: All studies that evaluated the impact of an intervention tool (i.e. not a pharmacy or nursing intervention) on the implementation of, uptake of, or adherence to a clinical practice guideline by an HCP in ambulatory care were included.
-Comparator: We selected studies that included at least one control group. Comparison groups included usual care, a similar guideline implementation intervention of differing intensity or duration than the main intervention group, or no intervention (receipt of the intervention at a different time than the intervention group, such as after data collection).
-Main outcome: Measures of guideline adherence by the HCPs. This may include self-reported adherence, direct observation, electronic monitoring, prescription review, chart review, or clinical outcomes.

### Exclusion criteria

–Trials based in an in-patient hospital setting

### Study selection and data extraction

Results from the literature search were directly imported into the online Covidence software package. This was used to screen results from the electronic databases, as well as the full-text articles indicated via the search. Titles and abstracts, as well as full-text reports, were screened independently by two reviewers(SM and JG). Disagreements were resolved by discussion.

Data extraction was completed in duplicate by two independent data extractors (SM and LM). The following information was extracted, where possible, from each study: author, country, year of publication, study characteristics (design, setting, population), disease process studied, participant characteristics, number of arms, description of intervention, comparison group, outcome measurement, results, and conclusion. Discrepancies between extractors were resolved through discussion and, when necessary, a third data extractor (JG). All data was managed using spreadsheets created for each extractor (Table 1).

**Table 1:** Table 1

	Study ID	Country	Study Type	Disease process studied	Participant description	Setting	Total number randomised	% Male participants	Arms	Intervention	Intervention description	Duration of intervention	Comparison	Primary Outcome Measure	Result	Conclusion
	ALERT *N*=11															
1	Adusumalli,2023	USA	cRCT	Dyslipidaemia	HCPs: Primary care physicians - internal or family medicine Patients who attended PCP during study period not previously prescribed a statin.	Primary Care	HCPs : 158 in 28 practices. 4131 patients	HCPs : 46.8% Patients : 51.3%	4	Electronic alert	Clinician nudge: Active choice prompt in EHR and monthly peer comparison feedback vs. Patient nudge : interactive text message 4 days before appointment vs. both clinician and patient nudge vs. usual care	6 months	Usual care	Initiation of a statin prescription by the end of the day of the primary care visit.	Clinician nudge increased statin prescribing alone (5.5 percentage points; 95% CI, 3.4 to 7.8 percentage points; *P* = .01) and when combined with the patient nudge (7.2 percentage points; 95% CI, 5.1 to 9.1 percentage points; *P* = .001).	Clinician nudge alone and when combined with a patient nudge significantly increased initiation of a statin prescription during primary care visits.
2	Adusumalli, 2021	USA	cRCT	Dyslipidaemia	HCPs : Attending Cardiologists. Patients: Visit with a Penn Medicine cardiologist during the study period and were candidates for statin prescribing by either the 2013 AHA/ ACC guidelines or NLA recommendations.	Secondary Care	82 Cardiologist. 11693 patients.	HCPs: 76.8% Patients : 57.7%	3	Electronic alert	Arm 1: Active choice - Interruptive best practice alert in EHR that required action. Arm 2: Passive choice – BPA embedded within patient encounter. Non interruptive. Did not require action. Arm 3 : Usual care	6 months	Usual care	Change in % of patients prescribed statin therapy at a dose that meets evidence-based guidelines (optimal dose).	No significant difference from control for passive choice (adjusted difference in percentage points, 0.2; 95% CI, −2.9 to 2.8; *P* = .86) or active choice (adjusted difference in percentage points, 2.4; 95% CI, −0.6 to 5.0; *P* = .08).	Passive choice and active choice interventions did not change statin prescribing among cardiologists.
5	Ashburner, 2018	USA	RCT	Atrial Fibrillation	Patients who had a diagnosis of AF, elevated stroke risk and were not currently prescribed oral anticoagulants.	Primary Care	HCPs: 175 2336 patients	Patients : 52% HCPs :41.7%	2	Electronic alert	Physician notification via email with clinical information, stroke and bleeding risk score and a link to educational material	3 months	Usual care	Proportion of patients prescribed oral anticoagulants at 3 months	No significantly difference between the notification (3.9%, 95% CI 2.8–5.3%, *n* = 38) and usual care (3.2%, 95% CI 2.4–4.2%, *n* = 44) arms (*p* = 0.37)	Electronic physician notifications did not increase anticoagulant prescriptions compared to usual care.
8	Coma, 2019	Spain	cRCT	Atrial Fibrillation, Hypertension, Cardiovascular Disease, Heart Failure	Primary care clinics. Patients aged over 14 years with at least one of 10 clinical situations: including hypertension, AF, cardiovascular disease and heart failure	Primary Care	269 primary care centres. 645,845 patients.	HCPs : Nor specified. 53.8% patients	4	Electronic alert	Electronic reminders were automatically displayed in a pop-up window when the HCP accessed the patient’s medical record. Pop-ups made a recommendation and where appropriate showed clinical values. Arm 1) Pop-up window Arm 2) Pop-up window with a schedule icon Arm 3) Pop-up window with a schedule icon plus a reminder filter settings option	6 months	Usual care – monthly feedback as usual	Resolution of electronic reminders (Clinically improvable condition for which it was generated was resolved).	The intervention led to a 20.6% improvement (OR 1.29, 95% CI: 1.25–1.34) in adherence to clinical recommendations.	Electronic point-of-care reminder systems can have a positive effect. Statistically significant differences were observed in all types of reminders.
11	Eckman,2023	USA	RCT	Atrial Fibrillation	Primary care clinicians in the University of Cincinnati Healthcare system. Patients with AF in whom a change to appropriate antithrombotic therapy – as determined by the Atrial Fibrillation Decision Support Tool (AFDST) - would result in a substantial gain (≥0.5 QALYs).	Primary Care	608 patients. 560 HCPs.	47% of patient cohort. HCPs not specified.	2	Decision support, Electronic Alert	Addition of best practice alert to the existing AFDST.	3 months	Access to AFDST embedded into EHR	Change to "appropriate anticoagulation" 3 months after randomisation	Effectiveness at 3 months following patients' index visit : 5% in usual care arm vs. 11% in intervention arm had their anticoagulation management changed to “appropriate therapy” (*p* = 0.02). Odds ratio of 2.31 (95%CI, 1.17–4.87).	BPA added to an already existent AF decision support tool, improved anticoagulation therapy utilisation among AF patients.
14	Ghazi, 2022	USA	cRCT	Heart Failure	HCPs providing care for HFrEF patients in outpatient internal medicine and cardiology practices affiliated with the Yale-New Haven Health System. Patients: Age > 18 years, LVEF ≤ 40%, not receiving all 4 classes of GDMT for HFrEF	Secondary Care	93 HCPs, 1310 patients	Patients: 69.3%, HCPs: Not specified	2	Electronic Alert	EHR- embedded BPA that triggered for eligible patients displaying clinical details, link to guidelines and highlighting if the patient was not on all classes of GDMT.	30 days	Usual care	Proportion of patients who had an increase in the number of prescribed GDMT classes at 30 days	The primary outcome was observed in 25.7% of the alert arm and 18.7% of the no-alert arm (adjusted relative risk: 1.41; 95% CI: 1.03-1.93; *P* = 0.03)	An alert that provided personalised recommendations for outpatients with HFrEF led to substantial increases in the use of GDMT.
17	Gupta, 2013	USA	RCT	Heart Failure	Patient: With ECG(s) obtained from January 2007 - July 2010 at the VA Palo Alto Health Care System and with LVEF ≤35%. HCPs: Primary care physicians & Cardiologists	Primary & Secondary Care	Patients: 89, HCPs: not specified.	Patients : 97%. HCPs : not specified.	2	Electronic alert	Clinical reminder stating potential eligibility for an ICD in the patient's EMR.	6 months	Usual care	Referral for consideration of defibrillator implantation < 6 months of randomisation	11 of 46 (24%) reminder patients met the primary outcome (referral for ICD evaluation) compared with 1 of 43 (2%) control patients (*P* = 0.004)	EMR-based clinical reminder to providers of patients with reduced LVEF improves the rates of referral for consideration of ICD implantation
19	Holt, 2017	UK	cRCT	Atrial Fibrillation	HCPs: Practices in South East and Central England. Patients: Diagnosis of AF, and fulfilling the eligibility criteria for an OAC.	Primary Care	HCPs: 47 practices Patients : 6429	Patients: 54.55%, HCPs: Not specified	2	Electronic alert	Use of Automated Risk Assessment for Stroke in Atrial Fibrillation (AURAS-AF) tool. A tool that could identify patients as eligible for but not using OAC and give a reminder to the clinician. HCPs prompted to indicate a reason if OAC not started.	6 months	Usual care	Proportion of patients eligible for OAC who were currently prescribed an OAC at the end of the 6 months intervention period.	The mean proportion of eligible patients prescribed OAC at six months was 66.3% in the intervention arm vs. 63.9% in the control arm. The adjusted mean difference (95% CI) was 1.21% (-0.72, 3.13), *P*=0.213.	No significant change in OAC prescribing occurred with the use of an automated software system
20	Kapoor, 2020	USA	cRCT	Atrial Fibrillation	HCPs: Cardiology or primary care providers taking care of at least 5 patients with AF with a CHA2DS2VASc score ≥2 and not currently on anticoagulation. Patients: With AF and a CHA2DS2VASc score of ≥2 with at least one clinic visit in the previous 12 months.	Primary Care & Secondary Care	HCP: 119, Patients: 5475	HCPs: 62.45%, Patients: 55.5%		Education, Dashboard, Electronic Alert, Feedback	Electronic profiling/messaging combined with academic detailing: Monthly reports of their anticoagulation percentage relative to peers for AF patients with elevated stroke risk. EMR-based messages shortly before an appointment with an anticoagulation-eligible but untreated AF patient. Invitation to an academic detailing session based on knowledge gaps discussed in provider focus groups.	5 months	Usual care	Change in % of eligible patients prescribed anticoagulation in the intervention and control groups over 6 months.	Patients not on anticoagulation at baseline: adjusted percent increase in the use of anticoagulation over 6 months was 5.2% (intervention) vs. 7.4% (control) *P*=0.21.	Combined intervention including dashboard feedback, electronic alerts and academic detailing did not increase anticoagulation use among patients with AF.
26	Mukhopadhyay, 2023	USA	cRCT	Heart Failure	HCPs: Cardiologists working in outpatient practices in the New York University Langone Health system. Patients: > 18 years, seen by a cardiologist during the study period, with an ejection fraction < 40%, and no active prescription for MRAs.	Secondary Care	HCPs: 180, Patients: 2211	HCPs: Not specified, Patients: 71.4%	3	Electronic Alert	Group 1: Automated EHR Alert - Alert on patient's chart with information related to MRA therapy, link to guidelines and option to prescribe or input reason not to prescribe Group 2: Automated EHR Message - An automated message each month in the EHR that linked to a list of patients who had been seen recently / due to be seen with all clinical details needed for MRA prescribing. Group 3: Usual care	6 months	Usual care	% of patients with newly prescribed MRA therapy from the initial alert or message date to the end of the study period.	New MRA prescribing occurred in 29.6% of patients in the alert arm (relative risk: 2.53; 95% CI: 1.77-3.62; *P* < 0.0001), 15.6% in the message arm (relative risk: 1.67; 95% CI: 1.21-2.29; *P* = 0.002), and 11.7% in the control arm.	An automated, patient-specific, electronic health record–embedded alert increased MRA prescribing compared to both embedded messages and usual care.
30	Piazza, 2023	USA	cRCT	Atrial Fibrillation	HCPs: Primary care and cardiovascular medicine physicians working in Brigham and Women’s Hospital group Patients : Aged > 21 years with AF and CHA2DS2− VASc score ≥ 2 who were not prescribed anticoagulation	Primary Care & Secondary Care	Patients: 798, HCPs: Not specified	Patients: 58.5%, HCPs: Not specified	2	Decision support, Electronic Alert	On screen notification (EPIC BPA) including the patient’s increased risk of stroke in AF, the lack of an active prescription for anticoagulation, and the indication for anticoagulant therapy. Option to prescribe / access guidelines/decline to prescribe with reason.	Follow up at 48 h and at 90 days after randomisation.	Usual care	The frequency of new orders for anticoagulation in alert versus control patients at 48 hours and 90 days post clinic visit.	Alert patients were more likely to be prescribed anticoagulation within 48 h of the clinic visit (15.4 % vs. 7.7 %, *p* < 0.001) and at 90 days (17.2 % vs. 9.9 %, *p* < 0.01).	Alert-based CDS increased the prescription of anticoagulant therapy in high-risk ambulatory care patients with AF.
	EDUCATION *N*= 14															
7	Collins, 2021	Tajikistan	cRCT	Hypertension	Adult patients (> 18 yrs) on the hypertension register who had attended in the past 12 months.	Primary Care	19 clinics. 1085 patients.	HCPs : Not specified 40.6% patients	2	Education, Decision support, practice facilitation	- Clinical protocols for hypertension and diabetes -In-person two-day training event - Supportive supervision visits - Paper-based clinical decision support tools - Quality improvement and management tools including quarterly monitoring visits using the HEARTS monitoring tool and direct support	12 months	Usual care	The proportion of hypertensive patients whose blood pressure was controlled ((SBP < 140mmHg and DBP < 90mmHg)	The proportion of hypertensive patients in the intervention group with blood pressure in normal range significantly improved from baseline to follow-up (OR 3.556, 95 % CI 2.219, 5.696); there was no significant change in the control group (OR 0.644, 95 % CI 0.370, 1.121) *p* < 0.001.	It is feasible to use routine, paper-based, clinical records to evaluate essential CVD interventions in primary health care in Tajikistan. Adapted WHO PEN/HEARTS guidelines in the context of a complex intervention significantly improved blood pressure control after 12 months.
13	Gattellari, 2020	Australia	cRCT	Atrial Fibrillation	Patients: Patients with AF who were not receiving anticoagulation or for whom management was challenging. HCPs: Random sample of GPs from across Australia whose caseload included elderly patients.	Primary Care	179 GPs. 590 patients.	Patients: 52%. HCPs: not specified.	2	Education, Decision support	Academic detailing: Educational materials, case discussion, summary of challenging cases including risk of stroke and safety messages. Expert decision support via written feedback on selected cases.	12 weeks	Academic detailing only.	Proportion of cases receiving anti-coagulant treatment post intervention.	Intervention group: 52.2% (*N* = 130) vs control group: 47.6% (*N* = 118). Difference not statistically significant: Adjusted Relative Risk = 1.11, 95% CI = 0.86 to 1.43, (*p* = 0.42.	Specialist feed-back in addition academic detailing did not increase anti-coagulant prescribing in Australian general practice.
15	Gulayin, 2019	Argentina	cRCT	Dyslipidaemia	HCPs: Physicians from 10 public PCCs. Patients: Aged 40 -74 years, with a history of CVD, high CVD risk, T2 diabetes or LDL-c level ≥190 mg/dL.	Primary Care	357 patients. 10 Primary care centres.	Patients : 36.4%. HCPs not specified.	2	Education	Education: (1) Intensive 2-day training workshop (2) Educational outreach visits x 3 (3) Mobile health application installed on the physician’s smartphones Support Tools: (1) Weekly SMS messages for study patients; (2) Onsite training to pharmacist assistants (3) Educational flyers	12 months	Usual care	Net change in LDL-c levels from baseline to 12 months	Mean LDL cholesterol difference from baseline: Intervention group: −8.7mg/dl (95% CI : −24.3, 6.8) vs. Control group : −8.0mg/dl (95% CI :−15.9, −0.2) Net difference −0.7 (95% CI :−18.1, 16.7) p value = 0.9366	No differences were observed in the mean LDL-c values between groups.
22	Kerfoot, 2014	USA	cRCT	Hypertension	HCPs: Primary care providers at 8 VA medical centres in the New England region with ≥half-time clinical commitment. Patients: Adult patients with ≥ 1 encounter with elevated BP (> 140/90 mm Hg).	Primary Care	111 clinicians. 14,336 patients.	HCPs: 34%, Patients: 94.4%	2	Education	Online spaced-education game where clinicians were e-mailed one question every 3 days. Posting of relative performance compared to peers.	12 months	Educational content via online posting	Time to BP target (< 140/90 mm Hg) in a hypertensive period (days).	After multivariate adjustment, the median time to BP target for all patients was 142 and 148 days for the SE game and control cohorts, respectively (*p* = 0.018).	An online SE game generated a modest but significant reduction in the time to BP target among their hypertensive patients.
23	Leupold, 2023	Germany	cRCT	Hypertension	HCPs: GPs accredited for the statutory health insurance system. Patients: Patients with uncontrolled practice BP (≥ 140/90 mmHg).	Primary Care	HCPs: 50 primary care practices. Patients: 636	Patients: 52.7%, HCPs: Not specified	2	Education	PIA : PC- supported case management of hypertensive patients to implement guideline-based hypertension therapy using a physician-defined and -supervised, patient-specific therapeutic algorithm. Secure patient app for communication, BP measurements, messages, guideline-orientated algorithms, eLearning, analysis and medication suggestions.	12 months	Usual care	BP control rate (BP < 140/90 mmHg) after 6–12 months.	BP control rate in the intervention group : 59.8% (95% CI: 47.4–71.0%) vs. control group : 36.7% (95% CI: 24.9–50.3%). Increased control rate by 23.1% (95% CI: 5.4–40.8%)	PIA-ICT system for hypertension management showed a significant improvement of BP control rates after 6–12 months.
31	Pimenta, 2014	Brazil	cRCT	Hypertension	HCPs: Primary care physicians in southeast Brazil who were engaged in the CME program Patients: Hypertensive patients who were > 45 years old for men, or > 55 years old for women, without previously diagnosed cardiovascular disease.	Primary Care	HCPs: 70, Patients: 467	HCPs: 36.6% Patients : 33.8%	2	Education	Educational workshop on hypertension, 1 outreach visit by a cardiologist, and 3 reminders via email.	3 months	Passive education	(1) Change in management of HTN that made the physician’s decisions more consistent with the guidelines (2) improvement in the prescription of hypertensive drugs (3) use of drugs that reduce the risk of cardiovascular disease	The active participation group outperformed the passive group: improved prescription of antihypertensive drugs (80% vs 51% ; *p* < .01), prescription of aspirin (18% vs 6%; *p* < .01) and hypolipidaemic drugs for high-risk patients (39% vs 21%; *p* < .01), dietary counselling (76% vs 61%; *p* < .01), guidance on cardiovascular risk (20% vs 3%; *p* < .01).	Multifaceted intervention improves treatment of hypertensive patients
32	Pouchain, 2013	France	cRCT	Hypertension	HCPs: GPs who were members of the French National College of Teachers in General Practice (CNGE) Patients with hypertension between 45-75 years with additional CV risk factors	Primary Care	HCPs: 335 Patients: 1832	HCPs: Not specified, Patients: 63.5%	2	Education, Decision support, Feedback	GPs attended a one-day training session on hypertension, were given an electronic blood pressure measurement device to ensure accuracy, and a leaflet on summarised targets and therapeutic strategies. They focused one consultation on hypertension and another on cardiovascular risk factors every six months for two years. They also received feedback at baseline and at one year on their patients’ clinical and biological parameters.	2 years	Usual care	Change in the proportion of patients achieving all of their therapeutic targets at two years: 3 targets in those without T2DM :BP ≤ 140/ 90 mmHg, LDL ≤ 3.36 mmol/l, and no smoking. 5 therapeutic targets for patients with type-2 diabetes: BP ≤ 130/80 mmHg, LDL ≤ 2.59 mmol/l, HbA1c ≤ 7%, no smoking, and a prescription for low-dose aspirin.	The proportion of patients achieving all of their therapeutic targets increased in both groups, but the increase was significantly higher in the intervention group: OR 1.89, 95% CI 1.09 to 3.27, *P* = 0.024.	Multifaceted intervention aimed at GPs increased the number of high risk hypertensive patients achieving therapeutic targets.
35	Siebenhofer, 2019	Germany	cRCT	Atrial Fibrillation, Cardiovascular disease	HCPs: GPs & HCAs in primary care practices that provide care to patients with statutory health insurance. Patients: > 18 years of age, had a long-term indication for OAC based on the guidelines valid at the time, and were prescribed coumarins, antiplatelet therapies, or DOACs.	Primary Care	HCPs: 52 GP practices (exact no. of HCPs not specified), Patients: 736	Patients: 55%, HCPs : 65.4%	2	Education	- Tools and guidelines for GPs and their practice teams - HCA Training Workshop - Phone call to GPs before after randomisation to provide with further information on case management - Quality circles to discuss the practical problems x 3 - Intervention practice teams received €50 per enrolled patient and assessment All practices were provided with the evidence-based “Anticoagulation” guideline for GPs	2 years	Usual care. Anticoagulation guidelines provided.	Combination of all thromboembolic events requiring hospitalisation and major bleeding complications.	Primary endpoint occurred in 40 (11.0%) intervention and 48 (12.9%) control patients (hazard ratio 0.83, 95% CI 0.55 to 1.25; *P* = .37).	This multi-faceted intervention did not show a statistically significant difference in the number of all thromboembolic events requiring hospitalisation and major bleeding complications.
36	Steyn, 2013	South Africa	cRCT	Hypertension	HCPs: HCPs working at CHCs with a minimum of 25 patients with diabetes and 35 with hypertension on their registers, Patients: $\underset{\_}{\ge }$ 15 years or older, at least four visits during the previous year for hypertension or diabetes, and having received treatment for these conditions at each visit.	Primary Care	HCPs: 18 clinics (number of HCPs not specified. Patients: 920.	HCPs: Not specified, Patients: 21.3%	2	Education	A structured record (SR), was placed in each patient’s folder which included guidelines, diagnostic algorithms, names and doses of medication. 3 outreach visits by a hypertension expert for education.	1 year	Usual care	Mean systolic and mean diastolic BP in all those with hypertension as measured at the end of the intervention period of 1 year.	BP increased from approx. 150/88 to 161/88 mmHg in intervention as in control groups (152/86 to 158/87 mmHg). Mean intervention effect (95% CI) SBP : 4.8 (–1.3, –10.9), DBP : 0.93 (–2.07, –3.93).	Educational outreach and SRs did not show any significant benefit in BP control.
37	Valk, 2020	Netherlands	cRCT	Heart Failure	HCPs: HCPs working in PCPs in Utrecht Patients: Adult patients with a confirmed diagnosis of heart failure.	Primary Care	HCPs: 30 PCPs (no. of HCPs not specified), Patients: 398	HCPs: Not specified, Patients: 47.5%	2	Education	Half-day training session on HF management.	6 months	Usual Care	The use of guideline-recommended HF medication in patients with HF at 6 months.	In patients with HFrEF (*N*=204), the use of ACEIs/ARBs decreased by 5.2% in the intervention group and by 5.6% in the control group [baseline-corrected OR 1.07 (95% CI 0.55–2.08)], beta-blocker use increased by 5.2% in the intervention group and 1.1% in the control group [baseline- corrected OR 0.82, (95% CI 0.42–1.61)], MRA use increased in the intervention group by 2.3% and in the control group by 1.1% [baseline- corrected OR 0.85, (CI 0.39–1.88). No clear differences in prescription rates of HF drugs between intervention and control group in those with HFpEF.	A half-day training session for GPs does not improve drug treatment of HF in patients with established HF.
39	van Riet, 2016	Netherlands	cRCT	Heart Failure	HCPs: GPs working in practices in the Zeist region in the Netherlands Patients with newly detected heart failure aged 65 or older.	Primary Care	HCPs: 30, Patients: 92	HCPs: Not specified, Patients: 47.8%	2	Education	Single half-day educational session.	6 months	Usual Care	Changes in the proportion of patients on diuretics and disease-modifying therapy, functional capacity (6MWT), health status and HF-related healthcare visits, referrals and hospitalisations	- There was no significant difference in medication prescription for HFrEF patients for the prescription of diuretics (57% in the intervention vs 60% in control group), ACE inhibitors/ARBs (72% in the intervention group vs 70% in the control), beta-blockers (57% in the intervention group vs 50% in control) or MRAs (none in both groups) at 6 months - For HFpEF, the percentage of patients in which medication was initiated or up titrated was similar in both groups. - Patients in the intervention group showed an improvement in functional capacity as measured with the 6MWT (mean difference 28 m, 95% CI 3 to 53), more often visited the GP (RR 1.8; 95% CI 1.3 to 2.5) and less often consulted a cardiologist (RR 0.6; 95% CI 0.3 to 1.1).	Training GPs in optimisation of drug treatment of newly detected HFrEF and HFpEF did not clearly increase HF medication, but resulted in improvement in walking distance.
41	Vinereanu, 2017	Chi na, Argentina, Brazil, India, Romania	cRCT	Atrial Fibrillation	HCPs: HCPs from cluster sites in each of the 5 participating countries with access to 40-90 of eligible patients. Patients: Patients ≥ 18 years with AF who with an indication for oral anticoagulation (CHA2DS2VASc score ≥ 2).	Primary care & Secondary Care	HCPs: 48 clusters (Number of HCPs not specified), Patients: 2281	HCPs: Not specified, Patients: 53%	2	Education, Feedback	Education for providers and patients, with regular monitoring and feedback.	1 year	Usual care	Change in the proportion of patients treated with oral anticoagulants from baseline assessment to evaluation at 1 year.	The proportion of patients on oral anticoagulation increased from 68% at baseline to 80% at 1 year in the intervention group (difference 12%) and from 64% to 67% in the control group (difference 3%), with an absolute difference in the change of oral anticoagulation use of 9·1% (95% CI 3·8–14·4) corresponding to an odds ratio of 3·28 (95% CI 1·67–6·44; adjusted p value=0·0002)	A multifaceted and multilevel educational intervention resulted in a significant increase in the proportion of patients treated with oral anticoagulants.
44	Weltermann, 2016	Germany	cRCT	Hypertension	HCPs: PCPs caring for hypertensive patients and who were equipped with a calibrated ABPM device, working in practices from the general medicine practice network of the University of Duisburg-Essen. Patients: Hypertensive patients aged ≥ 18 years with uncontrolled BP according to the European hypertension guidelines.	Primary Care	HCPs: 24 practices (exact number of HCPs not specified), Patients: 169	HCPs: Not specified, Patients: 56.3%	2	Education	Three CME sessions combining evidence-based information and practice implementation strategies.	5 months	Usual care. Delayed intervention	The primary outcome was BP control (ABPM < 130/80 mmHg) after 5 months.	The intervention had no effect on BP control (odds ratio 0.84 [95% CI 0.29–2.43]) and BP changes (interventional effect: systolic –2.48 mmHg [95% CI –7.24 to 2.29], diastolic –0.25 mmHg [95% CI 3.31 to 2.82]).	The intervention had no impact on BP control
46	Zwarenstein, 2016	Canada	cRCT	Hypertension	HCPs: All Ontario physicians with an active general practice Patients: Ontario residents aged over 66 years newly treated for hypertension during the study period	Primary Care	HCPs: 5179 Patients: 23508	HCPs: 77.3% Patients: 47.1%	6	Education	Messages advising the use of thiazides as the first-line treatment of hypertension were mailed to each physician in conjunction with a widely read professional newsletter. Physicians were randomised to receive differing versions of printed educational messages (PEM): 1. Informed only (no PEM/ no mention of thiazides) 2. Informed + “insert” (two-page evidence-based article) 3. Informed + atheorectical outsert (short, directive message stapled to the outside of the newsletter). 4. Informed + theory of planned behaviour (TPB) -based outsert 5. Informed + insert + atheorectical outsert 6. Informed + insert + TPB-based outsert	Intervention : 1 month 1 year follow up	Usual care	The percentage of patients aged over 65 and newly diagnosed with hypertension who were prescribed a thiazide as the sole initial prescription medication.	No intervention effect was detected. Thiazides were prescribed to 27.6 % of the patients who saw control physicians, 27.4 % for the insert, 26.8 % for the outsert and 28.3 % of the patients who saw insert + outsert physicians, *p* = 0.54.	Printed educational materials did not increase the prescribing of thiazide diuretics as first-line management of hypertension.
	DECISION SUPPORT *N*=9															
3	Arts, 2017	Netherlands	cRCT	Atrial Fibrillation	All patients with AF (both incidental and pre-existing) who had been in contact with their GPs during the length of the trial were included.	Primary Care	781 patients with AF, 39 GPs in 19 GP clusters.	Not specified	3	Decision support, Electronic alert	Intervention - Clinical Decision Support System (CDSS) integrated into EHR system Intervention 1 : Pop up notification with actionable recommendation. Could decline without justification. Intervention 2: Pop up notification with actionable recommendation. Could only be declined if justification documented. Control group : No messages	11 months	Usual care	Proportion of patients with AF treated in accordance with 2013 Dutch GP guidelines for AF.	No statistically significant difference between groups. Control: 50%, Intervention: 55% chisq *P* = 0.23.	A decision support system in general practice did not increasing adherence to antithrombotic guidelines for atrial fibrillation
6	Bajorek, 2016	Australia	cRCT	Atrial Fibrillation	General practitioners. Patients aged 65 years or older, diagnosis of AF as confirmed by the GP.	Primary Care	48 GPs, 393 patients.	HCPs : Not specified 54.5% of patients	2	Decision support	Computerised anti-thrombotic risk assessment tool (CARAT) – generated recommendation based on clinical information. GPs asked to review output when making a decision on therapy	12 months	Usual care	Antithrombotic therapy prescribing before and after the application of CARAT.	Use of antithrombotic therapy did not change from baseline (204/206; 99.0%), but the use of anticoagulants increased significantly (*P* = .02) from 89.3% (184/206) to 92.2% (190/206), and the use of antiplatelet therapy decreased (but not significantly [*P* = .20]) from 9.7% (20/206) to 6.8% (14/206). 9 (4.4%; 9/206) patients in the intervention arm and none in the control arm immediately changed their therapy (P = .003).	Tools such as CARAT can assist clinicians in selecting antithrombotic therapies, particularly in upgrading patients from antiplatelets to anticoagulants.
9	Cox, 2020	Canada	cRCT	Atrial Fibrillation	Healthcare Professionals: Providers in primary care practices in the Province of Nova Scotia. Patient participants: Adult patients with a confirmed diagnosis of AF.	Primary Care	203 HCPs. 1145 patients.	HCPs : Not specified 62% patients	2	Decision support	Access to a Web-based, point-of-care computerised CDS tool which generated alerts, dosing support, access to guidelines, clinical parameters and feedback.	12 months	Usual care	Composite of any AF-related ED visit or unplanned cardiovascular hospitalisation.	CDS use had no statistically significant effect on time-to-first-event for the primary outcome of AF-related ED visit or unplanned CV hospitalisation (*n* = 69, 12.7% usual care, *n* = 79, 13.4% CDS) (HR: 1.06 [95% CI 0.77-1.47], *P* = .713)	The CDS tool used did not show superior efficacy to usual care.
12	Eckman,2016	USA	cRCT	Atrial Fibrillation	Patients: non-valvular AF. HCPs: Physicians working in primary care network of University of Cincinnati	Primary Care	70 HCPs, 1493 patients	Patients: 54%. HCPs: Not specified	2	Decision Support, Education	Summary report highlighting patients not on appropriate therapy / patient-level treatment recommendations as per AFDST / Education sessions.	10 months	Education only	The proportion of patients with antithrombotic therapy that was discordant from AFDST recommendations at one year follow-up.	The proportion of patients whose treatment was discordant from AFDST recommendations dropped from 41.9% (626/1493) in 2014 to 40.6% (606/ 1493; *P* = .10) in 2015.	No significant improvement in discordant antithrombotic.
16	Guo, 2020	China	cRCT	Atrial Fibrillation	Patients > 18 years, newly diagnosed with AF with a CHA2DS2-VASc score > or = 2. HCPs: local doctors working at the 40 participating cluster hospitals in 40 cities across China	Secondary care	3324 patients. 40 clinical sites.	Patients: 62%, HCPs: Not specified	2	Decision support, Education	Mobile AF Application (mAFA) platform available to doctors & patients in the intervention group which included: clinical decision support tools, educational materials and patient involvement strategies.	12 months	Usual care	Composite of stroke/thromboembolism, all-cause death, and rehospitalisation at 6 months and 12 months.	Rates of composite outcome : mAFA intervention group 1.9% vs 6% in control group. HR: 0.39; 95% CI: 0.22 to 0.67; *p* < 0.001.	An integrated care approach supported by mHealth technology, reduces the risks of rehospitalisation and clinical adverse events among patients with AF.
21	Karlsson, 2018	Sweden	cRCT	Atrial Fibrillation	HCPs: All clinicians at primary care clinics using the Cambio EHR in the county of Ostergotland. Patients with AF.	Primary Care	43 primary care clinics. 14,134 patients.	Patients: 57.3%, HCPs: Not specified	2	Decision support, electronic alert	Clinical decision support (CDS) that produced an alert for physicians responsible for patients with AF and at increased risk for thromboembolism (according to the CHA2DS2- VASc algorithm) without anticoagulant therapy. Prompted to give a reason if deviating from guidelines.	12 months	Usual care	Proportion of patients eligible for stroke prophylaxis who were prescribed anticoagulant therapy 12 months	Increase in guideline adherence in the CDS (73.0%, 95% CI 64.6%–81.4%) versus the control group (71.2%, 95% CI 60.8%–81.6%, *p* = 0.013	CDS can increase guideline adherence for anticoagulant therapy in patients with AF.
25	McKie, 2020	USA	cRCT	Atrial Fibrillation, Heart failure and hyperlipidaemia	HCPs: Primary care providers. Patients: Patients with a diagnosis of heart failure, hyperlipidaemia or AF	Primary Care	HCPs: 109 (from 20 care teams), Patients: 16,310	HCP: Not specified, Patients: 38%	2	Decision support, Electronic Alert	The clinical decision support system (CDSS) displayed an alert in the medical record if there was a discrepancy between current and guideline-recommended treatment. Clicking the alert displayed the treatment discrepancy, risk scores, decision aids, educational material and recommended treatment.	7.5 months	Usual care	The % of discrepancies between pre-visit treatment and guideline-recommended treatment that were resolved within 7 days of the patient visit. (Restricted to medication changes.)	The advisory CDSS increased adherence to guideline-recommended treatment for heart failure (odds ratio [OR] 7.6, 95% confidence interval [CI], 1.2, 47.5) but had no impact in atrial fibrillation (OR 0.94, 95% CI 0.15, 5.94) or hyperlipidaemia (OR 1.1, 95% CI 0.6, 1.8).	CDSS improved guideline-directed treatment for heart failure but not for atrial fibrillation or hyperlipidaemia. Overall usage of the CDSS was low.
33	Prabhakaran, 2019	India	cRCT	Hypertension	HCPs: Primary care physicians and NCD nurses working in community health centres Patients: ≥30 years of age, had been diagnosed with hypertension : BP ≥ 140/90 mmHg or T2DM	Primary Care	HCPs: 40 CHCs Patients: 3698	Patients: 55.2%	2	Education, Decision support	mWellcare arm Education : Training on the current clinical management guidelines. Charts on management of these conditions on display Electronic Decision Support (EDS) system: Tablet used to collect patient data and this was used to generate a decision support recommendation for the physician Feedback : Monthly feedback report	12 months	Enhanced usual care	Differences in mean change (from baseline to 1 year) in SBP among participants with hypertension	A decline in SBP from baseline to study end was observed in each arm: EUC: −12.7 mm Hg vs. mWellcare, −13.7 mm Hg. ∆=−0.98; 95% CI, −4.64 to 2.67.	No incremental benefit of mWellcare over enhanced usual care in the management of hypertension.
38	van Doorn, 2018	Netherlands	cRCT	Atrial Fibrillation	HCPs: HCPs working in general practices in the region of Utrecht Patients: Adult patients diagnosed with AF	Primary Care	HCPs: 38 practices (exact no. of HCPs not specified), Patients: 2355	HCPs: Not specified, Patients: 52.2%	2	Decision Support	GPs received a list of all identified AF patients in their practice whose treatment was not according to 2013 Guideline Atrial Fibrillation (2nd revision) of the Dutch College of General Practitioners. Advised to follow the recommendation to optimise anticoagulant treatment in shared decision with the patient Reminder email 1 month later	3 years	Usual Care	Composite of stroke, TIA and/or thromboembolism	The incidence rate per 100 person-years of ischaemic stroke/TIA/TE was 1.96 in the intervention group and 1.42 in the reference group (hazard ratio (HR) 1.3, 95% C.I. 0.8–2.1).	A CHA2DS2-VASc based decision support did not result in a reduction in the incidence of stroke, TIA and / or thromboembolism.
	FINANCIAL INCENTIVES *N*= 3															
4	Asch, 2015	USA	cRCT	Dyslipidaemia	Primary care physicians. Patients aged 18 to 80 years with a 10-year Framingham Risk Score (FRS) of ≥ 20%, had coronary artery disease equivalents with LDL-C levels of 120 mg/dL or greater, or had an FRS of 10% to 20% with LDL-C levels of 140 mg/dL or greater	Primary care	340 PCPs and 1503 patients	HCPs : 62.6% Patients : 57.37%.	4	Financial incentives	All patients were sent an electronic pill bottle for statins. Each patient in the 3 intervention groups was assigned a quarterly goal to reduce LDL-C. Control Group: No goal-based incentives tied to outcomes for physicians or patients Physician Incentive Group: Physicians were eligible to receive up to $1024 annually per enrolled patient meeting LDL-C goals. Patient Incentive Group: Patients were eligible for up to $1022annually distributed through daily lotteries tied to medication adherence. Physician and Patient Incentive Group: As above but with half the amount of financial reward.	12 months	Usual care	Change in LDL-C levels at 12 months	Patients in the shared physician-patient incentives group achieved a reduction in LDL-C level statistically different from those in the control group (8.5 mg/dL; 95% CI, 3.8- 13.3, *P* = .002), those in the patient incentives group (8.6 mg/dL, *P* = .003) and the physician incentives group (5.7 mg/dL, *P* = .03) at 12 months.	Shared financial incentives for physicians and patients, but not incentives to physicians or patients alone, resulted in a statistically significant difference in reduction of LDL-C levels at 12 months.
28	Petersen, 2013	USA	cRCT	Hypertension	HPCs : Full-time primary care physicians from 12 hospital-based primary care clinics in five Veterans Affairs (VA) Networks. Patients: Patients with a diagnosis of hypertension.	Primary Care	HCPs : 125. 12 hospital sites.	HCPs: 55.8%, Patients: Not specified	4	Education, Financial incentives, Feedback	4 study groups: (1) physician-level (individual) incentives (2) practice-level incentives (3) physician-level plus practice-level (combined) incentives (4) no incentives (control)	20 months	Usual care	The number of hypertensive patients among a random sample who achieved guideline-recommended blood pressure control, received an appropriate response to uncontrolled blood pressure; and/or been prescribed guideline-recommended medications and the number who developed hypotension.	The adjusted change over the study in patients meeting the combined blood pressure/ appropriate response measure was 8.84 percentage points (95% confidence interval [CI], 4.20–11.80) for the individual-level, 3.70 (95% CI, 0.24–7.68) for the practice-level, 5.54 (95% CI, 1.92–9.52) for the combined, and 0.47 (95% CI, −3.12–4.04) for the control groups. The use of guideline-recommended medications or incidence of hypotension did not significantly change compared to controls.	Individual financial incentives, but not practice-level or combined incentives, resulted in greater blood pressure control or appropriate response to uncontrolled blood pressure.
29	Peterson, 2021	USA	cRCT	Hypertension, Dyslipidaemia	HCPs: HCPs billing for Medicare Part B services with a certified electronic health record. Patients: Patients with Medicare Parts A and B, aged 40 to 79 years, with no previous MI / stroke.	Primary Care & Secondary Care	HCPs: 330 organisations, exact number of HCPs not specified Patients : 112,352	HCPs : Not specified. Patients :55.7%	2	Education, Financial incentives, Feedback	The US Centers for Medicare & Medicaid Services (CMS) paid organisations in the intervention group for risk stratifying patients and reducing CVD risk among high-risk patients.	Measurements taken at 12 months. Trial ongoing.	Usual care	Initiating or intensifying statin or antihypertensive therapy within 1 year of enrolment, and LDL-C and systolic blood pressure reduction approximately 1 year after enrolment for high risk patients. Increase in CVD medications for medium risk CVD patients.	High-risk patients in the intervention group were more likely than control patients (8127 [37.3%] vs 4753 [32.4%]; adjusted difference in percentage points, 4.8; 95% CI, 2.9-6.7; *P* < .001) to initiate or intensify statins or antihypertensive medication. Mean systolic blood pressure was 1.2% lower in the intervention vs control groups (*P* = .003) and LDL cholesterol level was 2.0% lower (*P* = .003). In medium-risk patients, rates of initiation or intensification of statins or antihypertensive medication was higher in the intervention group within 1 year of enrolment (27.9% vs 24.8%; adjusted difference, 3.1 percentage points; 95% CI, 1.9-4.3).	Financial incentives can improve use of CVD medications
	DASHBOARD *N*= 2															
27	Patel, 2018	USA	cRCT	Dyslipidaemia	HCPs: Primary care physicians Patients: Patients who met guidelines for statin therapy but not previously prescribed statin therapy	Primary Care	HCPs: 96, Patients: 4774	HCPs: 50%, Patients: 55%	3	Dashboard, Feedback	Automated patient dashboard using active choice framing with and without peer comparison feedback on performance to nudge primary care physicians to prescribe guideline-appropriate statins for patients who were not previously receiving statin therapy.	2 months	Usual care	The change in the % of eligible patients prescribed a statin.	There was a significant difference in statin prescribing in the active choice with peer comparison arm (adjusted difference in percentage points, 5.8; 95% CI, 0.9-13.5; *P* = .008), but not in the active choice arm (adjusted difference in percentage points, 4.1; 95% CI, −0.8 to 13.1; *P* = .11).	An automated patient dashboard using nudges that combined active choice framing with peer comparison feedback led to significant increase in statin prescribing rates.
40	Verma, 2023	USA	RCT	Heart Failure	HCPs: Physicians, nurse practitioners, and clinical pharmacists working at a Greater Los Angeles VA primary care facility. Patients: Age of 18 years, with a primary diagnosis of HFrEF with a last documented LVEF ≤ 35%, an estimated eGFR ≥ 30 mL/minute, and optimisation potential score (OPS) of ≤ 5/ 10.	Integrated Care	HCPs: Not specified, Patients: 300	HCPs: Not specified, Patients: 98.7%	2	Dashboard	Dashboard-guided telehealth-based clinic (DASH-HF clinic): -Dashboard data + chart review -+/- decision to proceed with a telehealth review	3 months	Usual Care	OPS 6 months after the end of the intervention. This was a composite score of all active prescriptions and prescribed doses for each class of GDMT.	The composite OPS for the intervention group was 2.9 (SD = 2.1) and 2.6 (S = 2.1) for the control group; adjusted mean difference 0.3 (95% CI, −0.1 to 0.7).	A single-point intervention did not significantly improve use and dosing of GDMT for HFrEF patients.
	FEEDBACK *N* = 2															
10	Dormuth, 2012	Canada	cRCT	Dyslipidaemia, Cardiovascular Disease	Family physicians in British Columbia. Patients > 25 yrs who visited participating family physician during study period who were at risk of becoming new statin patients.	Primary Care	2725 Family physicians.	69.5% of physicians. Patients not specified	2	Feedback, Education	Education for Quality Improvement in Patient Care (EQUIP) Prescribing Portrait: Personalised prescribing data, educational evidence based messages and figures for total statin prescribing for secondary prevention in the province.	12 months	Delayed group	Incidence of patients newly treated with statins for primary prevention.	Statistically significant 6% relative risk reduction was observed in the first year after the intervention (adjusted RR 0.94, 95% CI 0.91–0.98).	Significant beneficial effect on new statin prescribing for primary prevention.
45	Willis, 2020	UK	cRCT	Atrial Fibrillation, Hypertension	HCPs: Working in NHS general practices in West Yorkshire who used the SystemOne computerised clinical system. Patients: Patients with hypertension or AF.	Primary Care	HCPs: 64 practices (number of HCPs not specified), Patients: 488,865	HCPs: Not specified, Patients: 50%	2	Education, Decision support, Feedback	Audit and feedback with quarterly reports including peer comparison and evidence-based clinical messages. Educational outreach delivered by pharmacists with follow-up support.	11 months	Each intervention arm (AF / HTN) acted as the control arm for the other intervention arm (AF / HTN) in the same trial.	The proportion of patients achieving the lowest appropriate BP target, and those with AF with a CHA2DS2-VASc score ≥ 2 prescribed anticoagulation.	BP control OR 1.05, 97.5% CI 0.96–1.16, *p* = 0.215; anticoagulation prescribing OR 0.90, 97.5% CI 0.75–1.09, *p* = 0.214	A multifaceted implementation package had no effect on improving BP control or OAC prescribing.
	PROTOCOL / ALGORITHM *N*= 2															
42	Wang, 2020	China	cRCT	Hypertension	Participants: Employees between 18-60 with a diagnosis of hypertension	Primary Care, Workplace	60 workplaces. 4548 participants.	HCPs : Not specified Participants : 82.8%	2	Education, Protocol/Algorithm	1. Workplace wellness programme which included CVD health education, health food education and available at work, tobacco cessation, physical activity & physical environment modification, stress management and health screening. 2. Guidelines-based hypertension management protocol which focussed on participants with hypertension and included a CHC intervention and monthly visits.	2 years	Workplace wellness programme and usual care.	The change in BP control rate (BP < 140/90 mm Hg), from baseline to 24 months.	BP control rate in control group : 44.0% vs 66.2% in the intervention group. Overall intervention effect: OR, 1.77; 95% CI, 1.58-2.00; *P* < .001.	A multicomponent intervention strategy was more effective in improving BP control than usual care.
43	Wei, 2017	China	cRCT	Hypertension	HCPs: Family doctors working in the 67 township hospitals with EHRs in central Zheijiang province. Patients: Patients aged 50 to 74 years who had hypertension with a 10-year CVD risk of ≥ 20% or T2DM.	Primary Care	HCPs: 67 cluster hospital townships (exact no. of HCPs not specified) Patients: 28130	HCPs: Not specified, Patients: 48.9%	2	Education, Protocol/Algorithm, Feedback	Case management guidelines, training, monthly performance monitoring meetings and patient support activities. This included treatment algorithms, medication recommendations, advice on lifestyle interventions and medication adherence.	12 months	Usual care	The prescribing and taking of recommended highly effective medications and BP readings at quarterly intervals over 12 months	Participants in the intervention arm had substantially improved prescribing rates of anti-hypertensives, statins and aspirin (*P* < 0.001), and had higher medication taking rates of aspirin and statins (*P* < 0.001). Drug-taking rates of two anti-hypertensives were similar for intervention and control arms at around (*P* = 0.151) and mean systolic and diastolic blood pressures were similar across both arms (*P* = 0.79 and *P* = 0.05, respectively)	A multifaceted package used by family doctors improved the prescribing of dual anti-hypertensives, statins and aspirin.
	PRACTICE FACILITATION = 3															
18	Huebschmann, 2012	USA	RCT	Hypertension	Adult patients aged 18 to 79 years who had elevated BP.	Primary Care	Patients: 591, HCPs: Not specified	Patients: 45.35%, HCP: not specified	2	Practice facilitation, Education, Electronic alert	An outreach coordinator raised patient and provider awareness of unmet BP goals, arranged BP-focused primary care clinic visits, sent electronic prompts to EHR and provided link to guidelines.	Duration of intervention : 3 months	Usual care	Change in BP from baseline to end of study (9-month post-randomisation period)	There was no significant difference in change in BP control in intervention vs usual care groups (-10.1 / -4.1 vs -9.1 / -4.5 mm Hg, *P* = .50 and 0.71 for systolic and diastolic BP, respectively.	This multifaceted intervention did not result in improved BP control.
24	Liddy, 2015	Canada	cRCT	Hypertension, Cardiovascular Disease, Dyslipidaemia	HCPs: Primary care clinicians in Eastern Ontario Patients: Patients > 40 years with CVD / at high risk of CVD / T2DM or CKD	Primary Care	HCPs: 182 (from 84 practices), Patients: 5292	Provider : 38.5%, Patient 48.9%	3	Practice facilitation	Practice Facilitation: - Regular meetings with a facilitator over the study period (13 visits) - Visits included: audit and feedback, goal setting, integrating evidence-based care guidelines, enhancing community linkages, self-management support, and delivery system redesign.	2 years	Usual care	Mean adherence to indicators of evidence-based care (IDOCC Guidelines for CVS Disease) measured at the patient level.	There was a 1.9 % (95 % CI: −2.9 to −0.9 %) and 4.2 % (95 % CI: −5.7 to −2.6 %) absolute decrease in mean adherence from baseline to intensive and sustainability years, respectively.	Practice facilitation did not improve adherence to evidence-based guidelines for cardiovascular disease in primary care practices.
34	Shelley, 2020	USA	cRCT	Cardiovascular Disease	HCPs: Clinicians from small practices in NYC. Patients: Patients for whom aspirin, blood pressure control, cholesterol management or smoking screening/cessation intervention was indicated.	Primary Care	HCPs: 291 sites (Exact no. of HCPs not specified) Patients : Not specified	Not specified	4	Practice facilitation	13 practice visits over 1 year consisted of: optimising EHRs to monitor data, intervention updates and patient education materials and redesigning workflow to facilitate integrating evidence-based practice.	12 months	Usual care	Number of at-risk patients who reached clinical goals for each of the 4 ABCS guidelines: (A) Aspirin use (B) BP control (C) Cholesterol management (S) Smoking composite: screened for tobacco use and received counselling or cessation intervention ABC Composite : Patients with a history of IHD who met targets for aspirin, BP and cholesterol management.	Smoking-related outcomes improved when comparing follow-up with the control period (incidence rate ratio=1.152, 95% CI = 1.072, 1.238, *p* < 0.001) and when comparing follow-up with intervention (incidence rate ratio = 1.060, 95% CI = 1.013, 1.109, *p* = 0.007). The other outcomes did not reach statistical significance.	Practice facilitation was associated with improvements in smoking-related outcomes only.

### Quality assessment

Two reviewers (SM and LM) independently conducted a risk-of-bias assessment using the Cochrane risk-of-bias tool for randomised controlled trials (RoB 2) [12]. Conflicts were resolved by a third reviewer (JG). The RoB2 Excel tool (August 2019 version) was used to implement the risk of bias assessment.

There were five domains assessed. Judgements were rated as low, with some concerns or high risk of bias. The overall risk of bias was assessed using signalling questions and the tool algorithm.

### Data synthesis and analysis

The included articles were subdivided by intervention type to facilitate discussion and narrative synthesis of the findings. Given the heterogeneity of the study design and outcome measurements, a meta-analysis was not planned.

## Results

### Studies identified

**Fig. 1:**
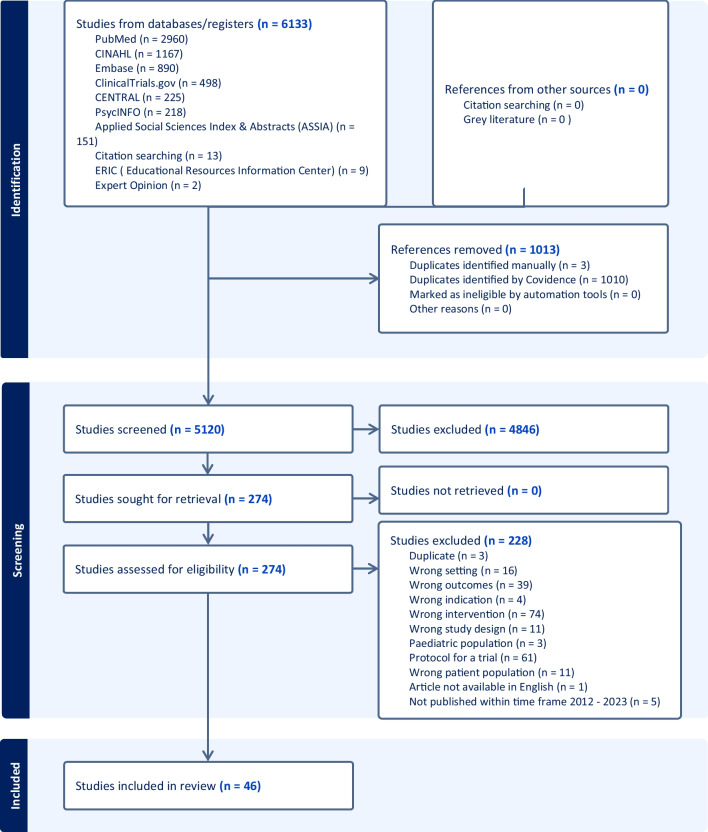
PRISMA Flow chart

A total of 6133 studies were identified from database searching. Following deduplication, 5120 records were screened, with 274 full texts assessed for eligibility, of which 46 were included in this review (Fig. 1, PRISMA flow chart).

### Description of included studies

We identified 46 studies that investigated the effect of HCP-targeted interventions. Most of these studies were conducted in North America (USA *N* = 19, Canada *N* = 4) and Europe (*N* = 12). There were 5 studies conducted in Asia, 2 in South America, 2 in Australia, and 1 in Africa. Only one study was conducted in more than one country [13].

Included studies examined interventions to improve adherence to AF guidelines (*N* = 14), HF guidelines (*N* = 6), hypertension guidelines (*N* = 12), dyslipidaemia guidelines (N = 6), CVD guidelines (*N* = 1) alone or a combination of these conditions (*N* = 7). The main categories of intervention used were education (*N* = 14), electronic alerts (*N* = 11), decision support tools (*N* = 9), financial incentives (*N* = 3), dashboards (*N* = 2), audit and feedback (*N* = 2), protocol/algorithm (*N* = 2), and practice facilitation (*N* = 3). Most of the included studies (*N* = 34) used a combination of approaches.

The majority of the included trials were cRCTs (*N* = 41) compared to RCTs (*N* = 5). The types of outcome measures used to assess adherence to guidelines were prescribing (*N* = 22), achieving disease targets, e.g. low-density lipoprotein cholesterol (LDL-C) targetor blood pressure (BP) target (*N* = 15), adherence to guideline recommendations (*N* = 4), clinical outcomes such as hospitalisation/stroke/transient ischaemic attack (TIA)/venous thromboembolism (VTE)/death (*N* = 4) and onward referral (*N* = 1). The length of the interventions with follow-up varied from 30 days [14] to 3 years [15].

## Results by intervention types

### Education

Fourteen studies used education as the main intervention type. Education was commonly used in conjunction with other interventions such as decision support [16, 17], practice facilitation [17], technology [18–20], and feedback [13, 20, 21]. The education component took various forms: workshops or in-person training [13, 17, 18, 21–26] provision of educational materials, [16, 27, 28], and online/eLearning/technology-based or use of digital tools [18–20].

Outcome measures included BP control [17, 19, 21, 22], prescription rates [26, 27], time to BP target [20], mean BP [28], LDL-c control [18], and correct oral anticoagulant use (OAC) [13, 16, 25].

Six studies demonstrated an improvement in the measured outcomes [13, 17, 19–21, 26], and eight did not [16, 18, 22–25, 27, 28]. Some noted an improvement in secondary outcomes only; Gulayin found no improvement in LDL-c control but observed improved statin dosing [18], the Weltermann study did not show any impact on BP control but did observe improved practice strategies [22], and the van Riet study did not improve prescription rates for those with HF but did show improvement in walking distances [24].

### Electronic alerts

Eleven studies investigated the use of electronic alerts, reminders, and notifications to improve adherence to clinical guidelines. The primary outcome measure for most of these studies was medication prescription [14, 29–36]. Eight of these studies reported an improvement in guideline adherence [14, 29, 30, 32, 35–38].

The two Adusumalli studies found increased rates of statin prescriptions with clinician nudges ± patient nudges [30] and best practice alerts (BPAs) [29]. The Coma study showed a 20.6% improvement in clinical recommendations with electronic reminders [38].

Eckman, Ghazi, and Mukhopadhyay also demonstrated improved prescription rates with BPAs in both AF [32] and HF [14, 35]. EHR reminders boosted implantable cardioverter defibrillator (ICD) referrals in those with HF and reduced left ventricular ejection fraction (LVEF) ≤ 35% in the Gupta study [37], and an alert-based clinical decision support (CDS) tool in the Piazza study was shown to increase OAC use [36].

However, some studies showed no improvement. The Ashburner study, which used electronic notifications, did not increase OAC prescriptions [31]. Similarly, both the Holt study [33] (on-screen reminders) and the Kapoor study (combined electronic profiling with academic detailing) did not significantly increase anticoagulation use [34].

### Decision support tools

Nine cRCTs focus on the implementation and evaluation of clinical decision-support tools. Adherence to recommended anticoagulant therapy in AF was a common outcome measure [39–43]. Other studies used clinical events such as stroke, thromboembolism, and hospitalisation as primary outcome measures [15, 44, 45]. The Prabhakaran study used the mean change in systolic BP as its primary outcome measure [46].

Overall, these studies generated varied results regarding the effectiveness of CDS tools. The McKie study demonstrated improved guideline-directed treatment for heart failure but not for AF or hyperlipidaemia [43]. Bajorek, Guo, and Karlsson also showed improvement in their primary outcomes [40, 42, 44], whereas results by Arts, Eckman, Cox, and Prabhakaran showed no significant effect [39, 41, 45, 46].

### Financial incentives

Three papers using a cRCT design investigated the impact of financial incentives on adherence to clinical guidelines [47–49].

These studies showed small but significant improvements in the outcomes measured. Asch showed that shared financial incentives for physicians and patients resulted in a statistically significant difference in the reduction of LDL-C levels at 12 months [47], whereas Petersen showed that physician-level financial incentives (but not practice-level or combined incentives) resulted in better BP control and appropriate response to uncontrolled blood pressure [49]. Similarly, Peterson showed that using the Million Hearts model, with financial incentives to participating organisations, increased the use or intensification of statins or antihypertensive medication and resulted in lower systolic blood pressure and LDL cholesterol [48].

### Dashboards

Patel and Verma investigated the use of dashboards as an intervention to improve adherence to heart failure [50] and statin [51] prescribing guidelines. These two studies had differing results. In DASH-HF, the dashboard did not significantly improve the optimisation potential score (OPS) or secondary outcomes such as hospitalisation and all-cause mortality [50], whereas in PRESCRIBE, there was a significant increase in statin prescribing rates in the active choice with peer comparison arm [51].

### Audit and feedback

Studies by Dormuth [52] and Willis [53] both used multifaceted interventions to improve prescribing practices in primary care settings with an emphasis on using audit and feedback. Dormuth used personalised prescribing portraits, whereas Willis used audit and feedback reports [52, 53]. These studies also included education and decision support.

The Dormuth study reported a 6% relative risk reduction in new statin prescriptions for primary prevention. This was based on an evidence-based message at the time the study was conducted which recommended a reduction in prescribing for primary prevention, particularly in women and elderly patients [52, 54]. Current guidance states that statin therapy for primary prevention may be considered in the elderly for those at high risk [55].

The Willis study did not have any significant impact on the primary outcomes of BP control or OAC prescription [53].

### Protocol/algorithm

Both the Wei and Wang papers are Chinese studies which used multifaceted interventions with a focus on protocol-based management [56, 57]. Wang’s analysis included a workplace wellness programme and a guideline-based hypertension management protocol with monthly visits [56]. The Wei study used a standardised community-based intervention package, including case management guidelines, treatment algorithms, and medication recommendations [57]. The workplace-based programme demonstrated an improvement in BP control [56], whereas the Wei study showed improved prescription rates of anti-hypertensive medications without a significant effect on BP control [57].

### Practice facilitation

Three papers investigate the role of practice facilitation in improving adherence to clinical guidelines; the Liddy [58] and Shelley [59] papers focus on CVD care, and Huebschmann [60] focuses on hypertension. These studies report varied results on the effectiveness of this intervention. Huebschmann noted reduced clinical inertia but no significant improvement in BP control [60], Liddy noted a small statistically significant decrease in adherence to guidelines [58], and Shelley reported an improvement in smoking-related outcomes (screened for tobacco use and received counselling or cessation intervention), but not in clinical CVD goals [59].

## Risk of bias

**Fig. 2:**
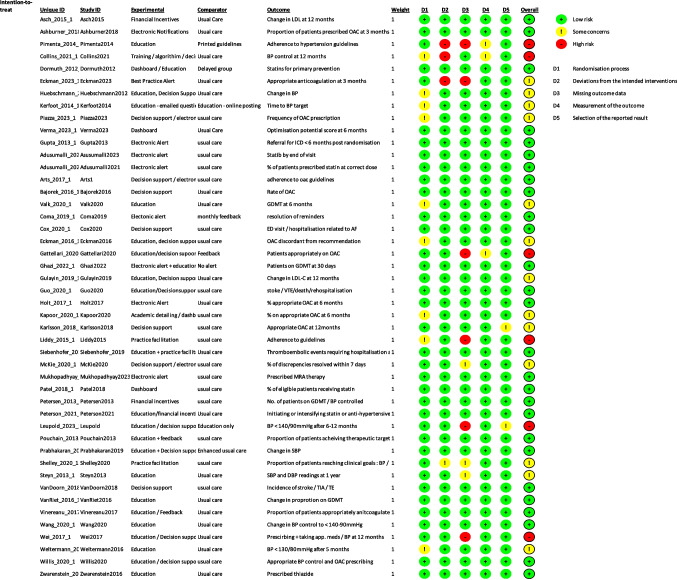
Risk of bias table

Most studies were at low-to-moderate risk of bias. Seven studies were rated as having a high risk of bias due to concerns over missing outcome data [16, 19, 41, 57, 58] and use of appropriate analysis [17, 26, 41] (Fig. 2: risk of bias table).

In five studies, it was unclear whether intention-to-treat analysis was applied [16, 17, 26, 41, 58]. Contributing factors included the exclusion of participants with missing outcome data or those lost to follow-up [16, 41, 58], the absence of a clear statistical analysis plan [17], and limiting analysis to only those who completed the intervention [26].

The Leupold study experienced disruptions due to the coronavirus pandemic, with several practices withdrawing or being lost to follow-up. Consequently, the study timeline was reduced from 12 to 6 months.^19^

Due to the reliance on routine reporting systems in the study by Wei et al., some data were missing [57]. The use of complete case analysis may have introduced bias, depending on the underlying cause of the missing data [57].

Given the nature of the included studies, blinding was not practical. The pragmatic nature of these trials aimed to simulate real-life scenarios and to test if implementing a certain intervention would improve adherence to guidelines.

## Discussion

This systematic review shows that interventions to improve adherence to CV guidelines use diverse approaches, with outcome measures varying from prescribing behaviours to disease control and clinical outcomes such as hospitalisations or death. While several studies showed improvement in guideline adherence, the overall effectiveness was mixed, with some studies showing improvements in secondary outcomes only. Some common challenges were highlighted, such as a short follow-up period, difficulties with implementation and engagement, and uncertainty over the generalisability of the findings.

Education, often combined with other methods, was the most common intervention but had inconsistent results. Multiple studies suggested that longer follow-up durations might be needed to detect significant changes in medication and prescribing practices or to determine whether the effects were sustained [16, 22, 23, 26]. Additionally, several papers noted difficulties with implementing interventions, particularly around engaging healthcare providers [13, 24, 28]. Studies conducted in specific regions, experienced academic centres, or resource-limited settings may not reflect outcomes that can be applied universally and raise concerns about the generalisability of the findings [13, 17, 20, 23, 24, 26, 28].

Financial incentives, though showing some modest improvements, raised questions about cost-effectiveness and sustainability, especially once incentives were withdrawn [47–49]. Similarly, dashboards and practice facilitation approaches faced challenges relating to implementation and clinician engagement [50, 51, 58–60].

Electronic alerts and decision support tools also demonstrated variable results. The effectiveness of these tools was often limited by low engagement rates and the phenomenon of alert fatigue, where clinicians became desensitised to frequent electronic notifications [29, 31, 32, 36, 39, 41, 43]. Interventions that integrated electronic alerts or decision support tools into the EHR system showed more favourable results [14, 32, 35–38, 40, 42]. Integration of these tools allowed alerts in real time, where immediate action could be taken [51]. This is in contrast to studies which did not demonstrate increased guideline adherence where the alerts or CDS tools were non-interruptive [29], came by email [15, 31, 34, 41], were available on an online platform [45] or audit/feedback results that came after the fact [16, 53]. As discussed, engagement with interventions was a limiting factor in many of the included studies. Using alerts in the moment, often with the patient present, creates the opportunity to review therapy and treatments, avoiding revisiting the chart or using up additional clinician time.

Multifaceted and multilevel interventions demonstrated superior outcomes compared to those with a single component. In particular, educational strategies that were part of a multifaceted approach were more successful at increasing guideline adherence [13, 17, 19, 21, 26, 44, 56, 57]. Educational interventions that were limited, e.g. half day training [23, 24], printed material [27], or limited CME sessions [22], were not shown to be effective. Recognising this, some of the authors advise that more intensive, multi-faceted interventions may be needed to meaningfully change clinical practice [15, 24, 31].

Systematic reviews on guideline adherence in HF and AF have also concluded that multifaceted interventions are most effective [61–63]. Meaningful dissemination of guidelines and clinical updates should not rely on educational strategies, which we found to be the most common intervention type. Providing additional educational resources or learning opportunities alone is not adequate to influence the behaviour of busy clinicians.

Overall, the findings suggest that while educational and technological interventions have potential, their success depends heavily on context, engagement, and sustained follow-up—indicating that multifaceted, integrated, tailored approaches may be more effective in changing clinical practice.

To address these challenges, there is a growing need to incorporate implementation science approaches into future research [64–66]. Implementation science focuses on understanding *what* works, *why* it works, and *how* interventions can be adapted to different settings [66]. This field emphasises evaluating contextual factors, stakeholder engagement, and the mechanisms by which interventions achieve their effects [66]. For example, the European Society of Cardiology has made implementation a key goal of their latest AF guideline and is running an RCT which seeks to improve guideline adherence in parallel with guideline production [5].

## Strengths and limitations

There are several limitations in this review. Given the heterogeneity in types of intervention and outcomes reported in the included trials, narrative synthesis of the data was difficult and meta-analysis was not possible. Different studies used different guidelines depending on the country or period in which the study was conducted; some of these guidelines have since changed. We acknowledge that guideline adherence is a surrogate marker for improved clinical outcomes. Our review included studies available in the English language; therefore, some relevant studies may have been missed.

Despite these limitations, this review is comprehensive in providing an up-to-date assessment of all types of interventions aimed at HCPs to improve adherence to guidelines across five disease processes. All screening, data extraction, and risk of bias assessment were completed by two trained reviewers to ensure reproducibility.

## Conclusion

In conclusion, this systematic review demonstrates that a variety of interventions aimed at healthcare professionals can potentially improve adherence to cardiovascular guidelines, but their overall effectiveness remains mixed. While education-based strategies were the most common, multifaceted approaches that combined education with other methods, such as decision support tools, electronic alerts, and financial incentives, showed more consistent success. Significant challenges, including short follow-up periods, difficulties with implementation, engagement, and limited generalisability of findings, were noted across many studies.

With more guidelines and increasing volumes of information in healthcare, effective implementation has become as crucial as guideline development itself. Without this focus, the gap between best practices and real-world application is likely to widen, limiting the potential benefits of these guidelines for improving patient care and outcomes.

## Supplementary Information

Below is the link to the electronic supplementary material.

Supplementary file1 (DOCX 15 KB)

## Data Availability

No new data were generated or analysed in support of this research.
