# Supplementary material for: Interventions to improve adherence to clinical practice guidelines when treating cardiovascular disease: a systematic review
Source: Ir J Med Sci. 2025 Sep 24;194(6):2047–77. doi: 10.1007/s11845-025-04057-5 (PMC12769678; doi:10.1007/s11845-025-04057-5)
Supplement: Supplementary file 1 — Supplementary file1 (DOCX 15 KB) [file 11845_2025_4057_MOESM1_ESM.docx]

**Search Strategy**

**PubMed**

“Primary Care” OR “primary health care” OR “primary healthcare” OR “General Practice” OR GP OR “Integrated Care” OR “Ambulatory Care” OR “ambulatory patient*” OR “Out Patients” OR outpatient* OR “out-patient*” OR “Family Medicine” OR “Family Practice” OR “clinic visit*” OR “nurse-led care” OR “nurse led care” OR “primary medical care” OR community OR home OR (("Physicians, Primary Care"[Mesh]) OR "General Practice"[Mesh]) OR "Ambulatory Care"[Mesh] OR "Primary Health Care"[Mesh]OR "Outpatients"[Mesh] OR

Hypertensi* OR “High blood pressure” OR “elevated blood pressure” OR "Hypertension"[Mesh] OR

Dyslipid?emia OR Hypercholesterol* OR “High cholesterol” OR “high cholesterol level*” OR “Elevated cholesterol” OR hyperlipid* OR Hypercholesterolemia[MeSH Terms] OR Dyslipidemias[Mesh] OR

“Atrial Fibrillation*” OR AF OR “A Fib” “atrial flutter” OR “a flutter” OR “paroxysmal atrial fibrillation” OR “permanent atrial fibrillation” OR “persistent atrial fibrillation” OR Atrial Fibrillation[MeSH Terms] OR

“Heart Failure” OR HFrEF OR HFpEF OR “ heart failure with reduced ejection fraction” OR “heart failure with preserved ejection fraction” OR “systolic heart failure” OR “diastolic heart failure” OR “Congestive heart failure” OR “congestive cardiac failure” OR “right sided heart failure” OR “right-sided heart failure” OR “left sided heart failure” OR “left-sided heart failure” OR "Heart Failure"[Mesh] OR

“Ischaemic heart disease” OR “ischemic heart disease” OR “Cardiovascular Disease” OR “Coronary Artery Disease” OR IHD OR CVD OR CAD OR “coronary heart disease” OR “ coronary atherosclerosis” OR “ coronary arteriosclerosis” OR angina* OR isch?em*OR “myocard* infarct*” OR “myocardial ischemia” OR

"Coronary Artery Disease"[Mesh] OR "Myocardial Ischemia"[Mesh]

AND

“Adherence to guidelines” OR uptak* OR “implementation of guideline* “OR Adheren* OR implement* OR Compliance OR “institutional adherence” OR “policy adherence” OR “Policy compliance” OR “protocol compliance” OR “Reference standard” OR “Physician compliance” OR “doctor adherence “ OR “guideline adherence” OR “provider compliance” OR “guideline uptake” OR “guideline use” OR adopt* OR "Guideline Adherence"[MeSH Terms] OR "Practice Patterns, Physicians'"[Mesh] OR "Diffusion of Innovation"[Mesh:NoExp]OR "Drug Prescriptions/standards"[Mesh] OR "Primary Health Care/standards"[Mesh]

AND

“practice guideline*” OR “Clinical Practice Guideline*” OR CPG OR Guideline* OR "Practice Guidelines as Topic"[Mesh] OR "Practice Guidelines as Topic/standards"[Mesh]

AND

randomized controlled trial[Publication Type] OR randomized[Title/Abstract] OR
placebo[Title/Abstract] OR "Randomized Controlled Trials as Topic"[Mesh]
